# Development and validation of the synthetic index of palliative care integration into primary health care

**DOI:** 10.1186/s12913-026-15191-w

**Published:** 2026-07-23

**Authors:** Carolina Muñoz Olivar, Juan Sebastián Gomez Quintero, Fernando Pastrana Rendon, Carla Taramasco Toro, Miguel Antonio Sanchez-Cardenas, Carlos Javier Avendaño-Vásquez

**Affiliations:** 1 PhD Program in Health Sciences, Faculty of Medicine, Antonio Nariño University, Bogotá, Colombia; 2 Center for Cancer Prevention and Control (CECAN), Santiago, Chile; 3 Faculty of Engineering, Andrés Bello University, Santiago, Chile; 4 Faculty of Engineering, Institute of Technologies for Innovation in Health and Well-Being, Andrés Bello University, Viña del Mar, Chile; 5 Faculty of Nursing, El Bosque University, Bogotá, Colombia; 6 Escuela de enfermería, Universidad Industrial de Santander, Bucaramanga, Colombia; 7 Faculty of Nursing (Postgraduate Programs), Andres Bello University, Santiago, Chile

**Keywords:** Palliative Care, Primary Health Care, Public Health, Indicators, Healthcare indicators

## Abstract

**Background:**

Integrating palliative care (PC) into primary health care (PHC) is essential to achieve universal health coverage and equity. However, most countries lack concise and validated tools to monitor this process. This study developed and validated a composite index to measure the degree of PC integration into PHC at the departmental level in Colombia.

**Methods:**

An ecological, cross-sectional study was conducted across 33 territorial units in Colombia (32 departments plus Bogotá D.C.) in 2024. Eight of 15 candidate indicators were retained to construct the index. Each indicator was normalized using a Min–Max linear transformation [0–1] and analyzed through principal component analysis with varimax rotation. The Synthetic Index of Palliative Care Integration into Primary Health Care (SIPCI) was built using a hierarchical weighting scheme combining domain-level and within-domain weights. Stability was assessed through jackknife leave-one-out, alternative normalizations methods, and four robustness scenarios including Monte Carlo perturbations. External validity was examined through correlations with socioeconomic and service-related variables.

**Results:**

SIPCI scores ranged from 0.04 to 0.71 (mean 0.38, SD 0.13). Overall, 52% of departments showed low or medium-low integration, while 48% showed medium-high or high integration. A clear spatial gradient was observed, with higher integration in central departments and Bogotá, and lower integration in Amazon and Orinoquia regions. Sampling adequacy was good (KMO = 0.742). Information loss was low (NMSE = 0.13; 0.10 after indicator reduction). Leave-one-out analyses showed strong concordance with the baseline index (Spearman * > 0.94*; Kendall * > 0.86*). Five subcomponents were identified, which rural accessibility and undergraduate PC training contributing most. The correlation between SIPCI and departmental income was positive but moderate, indicating that economic capacity may facilitate, but does not determine integration.

**Conclusions:**

The SIPCI provides a reproducible, system-level measure for monitoring and benchmarking territorial patterns of palliative care integration within primary health care in Colombia. The findings describe marked geographic heterogeneity and support the use of SIPCI for routine monitoring and planning, without implying causal mechanisms or patient-level effects. Although the index showed good internal coherence and robustness, further studies may help strengthen its construct validity and assess its applicability in other settings.

**Supplementary Information:**

The online version contains supplementary material available at https://doi.org/10.1186/s12913-026-15191-w.

## Background

Primary Health Care (PHC) is recognized by the World Health Organization (WHO) as the cornerstone of health systems and a public health strategy to achieve Universal Health Coverage (UHC) and the Sustainable Development Goals (SDGs) [1, 2]. Within this strategy, palliative care (PC) is considered an ethical responsibility of health systems and an essential function of PHC [3, 4]. The integration of PC into PHC ensures that people with serious or life-limiting conditions receive continuous, person-centred, and equitable care across all stages of life and all levels of the health system [5].

The Pan American Health Organization (PAHO) has emphasized that PHC is not limited to the first level of care but rather represents a comprehensive approach that coordinates the full range of health services, from health promotion to specialized interventions. The “30–30–30 PHC for Universal Health” commitment, launched in 2019, urges countries in the Americas to reduce access barriers by 30%, allocate at least 6% of GDP to health, and invest 30% of public expenditure in first-level care to strengthen integrated service networks [6, 7]. In this framework, integrating PC within PHC is fundamental for ensuring that equity, accessibility, and continuity become operational realities rather than policy aspirations.

Despite global recognition of PC as a public health priority, its development and integration remain highly uneven across regions. In Latin America, only a few countries have achieved generalized provision of PC (Global Development Level 3b), and significant gaps persist between urban and rural areas [8]. Colombia, an upper middle–income country with a Gini index of 53.9 in 2023 [9], illustrates these disparities. Although national legislation and public policies support PC as part of the essential health benefits, service availability and human resources remain concentrated in large urban centres, leaving peripheral regions underserved [10, 11].

Assessing the degree of PC integration into PHC requires a multidimensional approach that extends beyond service counts or financial inputs. It should include indicators reflecting system capacity, policy implementation, professional training, and equity in access and continuity of care [2, 12]. Composite indicators can capture this complexity by summarizing multiple dimensions into a single, transparent measure. When properly designed, they enhance comparability and support evidence-informed policymaking, but if poorly constructed, they can mislead interpretation or promote simplistic conclusions [13, 14].

This study addresses this gap by developing and validating the Synthetic Index of Palliative Care Integration into Primary Health Care (SIPCI), a composite measure designed to quantify the extent of integration across Colombia’s departments. The SIPCI combines palliative care development indicators and PHC system metrics to provide a comprehensive, context-sensitive evaluation of how effectively PC is embedded within the broader PHC strategy.

In this study, integration of palliative care into primary health care is conceptualized as a multidimensional, system-level capacity through which palliative care resources, services, competencies, and organizational processes become embedded within primary health care structures and functions. Accordingly, integration is operationalized through dimensions reflecting: (i) workforce training and availability of essential resources, (ii) provision of palliative care services and population access, (iii) territorial and rural accessibility, (iv) continuity and responsiveness across levels of care, and (v) contextual conditions that influence the need for and delivery of palliative care within PHC systems.

From this perspective, these dimensions are not viewed as isolated determinants or outcomes, but as observable manifestations of the extent to which palliative care has been incorporated into primary health care. This conceptualization was informed by the WHO framework for palliative care development, the WHO–UNICEF Primary Health Care Measurement Framework, and two complementary national studies. The first identified and prioritized indicators of palliative care integration through a modified Delphi process supported by expert stratification procedures and self-organizing maps, while the second explored how experts conceptualized integration through qualitative analysis of expert comments. Together, these studies contributed to the conceptualization of integration as a system-level construct encompassing workforce preparedness, service availability, accessibility, continuity of care, policy implementation, and health system responsiveness within primary health care settings [15, 16].

Accordingly, SIPCI is intended as a departmental-level monitoring tool to describe and compare territorial patterns of palliative care integration within PHC; it is not designed to evaluate clinical outcomes, rank providers, or support causal inference.

## Methods

### Study objective

The objective of this study was to design and validate a synthetic indicator to assess the degree of integration of PC into PHC in Colombia at the departmental level in 2024.

### Study design

We conducted an observational, ecological, and cross-sectional study. The units of analysis comprised all territorial entities of Colombia, including 32 departments and one capital district. All territorial units were eligible and included; no exclusions were made. This configuration represented a complete national census rather than a sample. All data corresponded to the year 2024, the most recent period with complete departmental information available.

Accordingly, the unit of inference is the department (health system level); therefore, all results should be interpreted at the territorial level and do not imply individual-level effects or causal relationships.

**Fig. 1:**
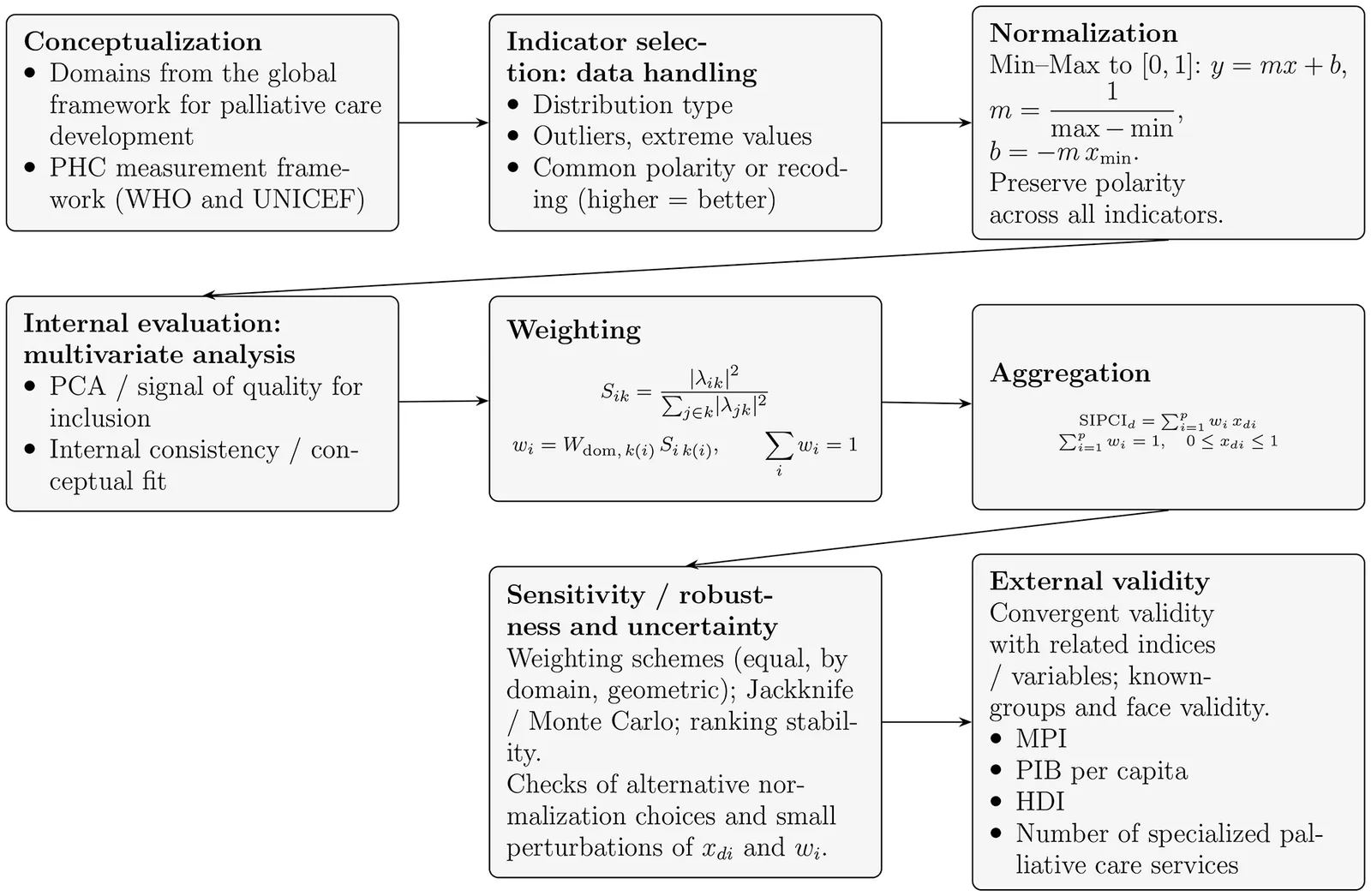
Methodological pipeline for the synthetic index of palliative care integration into primary health care (SIPCI). *Legend:* Methodological diagram for the construction of the SIPCI, based on the OECD framework for composie indicators. The figure presents the analytical stages of the process, from conceptualization and indicator selection to external validity assessment. Each box summarizes the key operations performed in that stage. The figure shows the methodological design only; no empirical results are included. **Abbreviations**: PHC: primary health care; PCA: principal component analysis; MPI: multidimensional poverty Index; GDP: gross domestic product; HDI: human development index

The methodological process followed the OECD/European Commission–JRC handbook for constructing composite indicators [14]. The analytical flow included conceptualization, indicator selection, normalization, multivariate analysis, weighting, aggregation, sensitivity and robustness assessment, and external validation. Figure 1 illustrates the overall pipeline.

Reporting adhered to the STROBE guidelines for observational studies [17], which provide a peer-reviewed reporting standard for observational research. This was complemented by the authors’ Methodological Appraisal and Credibility Assessment Checklist (MACA), an openly available methodological transparency tool, used to document and systematize key analytical decisions rather than to guide the construction of the index itself [18].

### Conceptualization

Integration of PC into PHC was understood as the effective incorporation of palliative principles and services within the essential components of PHC, ensuring continuity, accessibility, equity, and quality of care throughout the life course. This approach seeks to articulate health care across levels and service modalities, in both urban and rural settings, to provide a comprehensive and coordinated response to the palliative needs of the population.

Domains and subdomains of the indicator were based on:

- The global framework for palliative care development proposed by the WHO, which addresses the system-level components required for PC development, including national policy and planning, education and training, availability of essential medicines, service delivery, research and monitoring, public awareness, and progress tracking [12].
- The WHO/UNICEF conceptual framework for PHC measurement and monitoring, which organizes indicators across health system determinants (structures and inputs), service delivery (processes, outputs and quality), and health system objectives (outcomes and impact). The framework also recognizes three strategic and operational levers that strengthen PHC: integrated health services, multisectoral policy and action, and empowered people and communities [2].

### Indicator selection and data handling

The main outcome was the Synthetic Index of Palliative Care Integration (SIPCI), constructed from normalized quantitative indicators grouped in structural, service, and outcome domains.

Partial indicators were derived through a sequential mixed-methods process. An initial pool of candidate indicators was identified during a pre-Delphi stage and subsequently evaluated through a modified two-round Delphi study supported by expert stratification procedures and Self-Organizing Maps (SOM) to identify representative and discriminative indicators of palliative care integration within primary health care [15, 19]. A complementary qualitative analysis of expert comments was then conducted to refine the conceptual framework and indicator set, providing additional insight into how integration was understood and operationalized by experts [16]. Only quantitative indicators with departmental data were included in SIPCI construction; indicators available only at the national level, lacking territorial variability, or not operationalizable at the departmental level were excluded.

Variables were inspected for distributional properties, extreme values, and polarity consistency (all aligned as “higher = better”). No missing data were identified, and outliers were retained as they represented real observations. For multivariate analysis, binary (dichotomous) variables were also excluded, retaining only continuous quantitative indicators.

All retained indicators were grouped a priori into structural, service, and outcome domains, representing system determinants, service delivery processes, and system-level outcomes related to palliative care integration within PHC.

It is important to note that SIPCI combines indicators reflecting system capacity and readiness, such as workforce training, service availability, and access to essential medicines, with indicators reflecting contextual need or demand, such as the burden of chronic conditions requiring palliative care. Therefore, higher SIPCI values should not be interpreted as indicating better performance or higher quality of care, but rather as higher measured levels of system integration and responsiveness in the context of population needs.

### Normalization

To ensure comparability across heterogeneous indicators “not adding apples and oranges” [14], we applied linear Min–Max normalization to *[0,1]*:

$$y = m x + b,\quad m=\frac{1}{\max-\min},\quad b=-m\,x_{\min}.$$

Most variables were non-normally distributed (Shapiro Wilk, *p < 0.001*), which supported using a linear, order-preserving transformation while maintaining common polarity (higher values indicate better performance). For indicators where higher values reflected greater population need or disease burden rather than system capacity, values were inverted prior to normalization so that higher normalized values consistently indicated higher levels of palliative care integration at the system level.

### Internal consistency assessment

Before PCA, internal consistency was checked using:

- **Spearman correlations:** to identify redundancy between indicators by detecting highly correlated pairs (an *operational* threshold $\rho \ge 0.82$ was used to reduce redundancy while preserving information);
- **Kaiser–Meyer–Olkin (KMO):** statistic to assess whether indicators share sufficient common variance to justify the use of principal component analysis (variables with individual KMO * > 0.5* were reviewed conceptually);
- **Variance Inflation Factor (VIF):** to avoid excessive weighting of indicators conveying overlapping information by ruling out severe multicollinearity (VIF * > 10*).

Only indicators meeting statistical and conceptual criteria were retained for PCA.

### Dimensionality reduction and weighting

#### Principal component analysis (PCA)

PCA was used to concentrate multidimensional information into a reduced set of components explaining at least 85% of cumulative variance. We then applied orthogonal Varimax rotation to enhance interpretability and assigned each indicator to the component where its absolute loading was highest. Indicators were selected using a global 85th percentile threshold on the absolute rotated loadings; duplicated indicators across components were kept only in the component with the highest loading. The 85th percentile loading threshold was applied to retain the most informative indicator(s) per component, ensuring that each retained indicator was strongly associated with a single latent dimension. This criterion helped avoid double weighting of indicators conveying overlapping information when loadings exceeded the threshold across multiple components. As a result, a parsimonious set of indicators was obtained while preserving most of the information relevant for routine monitoring and territorial comparison.

#### Representativeness and reconstruction error

We computed communalities ($h_i^2$) and the normalized mean squared error (NMSE) of reconstruction to quantify information loss after dimensionality reduction. Since no universal NMSE cut-offs exist for PCA, we defined internal, operational ranges for comparative purposes: * < 0.10* (very low, ideal), 0.10–0.30 (low, acceptable), 0.30–0.70 (moderate, review with theory/weights), 0.70–1.0 (high, potential exclusion), * > 1.0* (very high, exclusion unless strong theoretical justification). These ranges are informed by guidance on stability and information loss in composite indicators and PCA reconstruction error [14, 20, 21].

#### Weighting

We used a two-level hierarchical scheme:

- **Domain weights:** the proportion of variance explained by each rotated principal component, rescaled to sum to 1;
- **Within-domain weights:** the squared absolute loading of each indicator, normalized within its component.

Thus, for indicator *i* in domain/component *k*:

$$\begin{gathered} {S_{ik}} = \frac{{|{\lambda _{ik}}{|^2}}}{{\sum\limits_{j \in k} | {\lambda _{jk}}{|^2}}},\quad {w_i} = {W_{{\mathrm{dom}},{\kern 1pt} k(i)}}{\mkern 1mu} {S_{i{\kern 1pt} k(i)}}, \hfill \\ \quad \sum\limits_i {{w_i}} = 1. \hfill \\ \end{gathered} $$

where $\lambda_{ik}$ represents the rotated loading of indicator *i* on component *k*, $S_{ik}$ is the normalized squared loading within each component, and $W_{\mathrm{dom},\,k}$ is the total weight assigned to the corresponding domain or component.

We verified that the weights sum to 1 and that final SIPCI scores lie within *[0,1]*.

No confounding adjustment was required, as the analyses did not estimate causal exposure–outcome relationships.

### Aggregation

We used a linear weighted aggregation, multiplying each normalized indicator by its weight and summing to obtain the departmental score. This approach preserves proportionality and allows direct comparisons, in line with OECD recommendations for composite indicators.

$$\mathrm{SIPCI}_d = \sum_{i=1}^{p} w_i\,x_{di} \quad \sum_{i=1}^{p} w_i = 1, \quad 0 \le x_{di} \le 1$$

Spatial and radar visualizations were produced in Python (JupyterLab) to display departmental SIPCI scores and indicator profiles by integration quartiles. All code and data are available in the Mendeley Data repository (DOI: https://doi.org/10.17632/dg8btkknvj.1) [22].

To further explore territorial patterns, departments were also grouped according to the seven geographic and functional *nodes* proposed by León et al. [23], which cluster the 33 territorial units of Colombia into seven regional groupings: Pacific, Caribbean, Amazon, Orinoquia, Central, Northeast, and Bogotá. These regional node analyses were descriptive and exploratory; no inferential subgroup testing was performed

### Sensitivity, robustness, and uncertainty analyses

We examined index stability under structural and methodological changes through:

- **Jackknife sensitivity:** exclusion of one indicator at a time, comparing each variant with the base index using Spearman and Kendall correlations and mean absolute ranking error (MAE).
- **Structural robustness (four scenarios):** R1 (equal weights across all indicators); R2 (equal weights by domain, with within-domain allocation by squared loadings); R3 (geometric aggregation of domain subindices, weighted by post-rotation explained variance); R4 (Monte Carlo simulations, 1000 iterations, $\pm 20\%$ perturbations to baseline weights), summarizing the 5th, 50th (median), and 95th percentiles of the Spearman correlation with the base index;
- **Alternative normalizations:** z-score to *[0,1]*, robust Min–Max, median/IQR to *[0,1]*, and rank/percentile scaling; we quantified changes in correlations and MAE of rankings.

The analysis was ecological and did not include subgroup or individual-level analyses

### External validity

External validity was assessed via Pearson correlations between SIPCI and theoretically related variables: Gross Domestic Product (GDP) per capita, Multidimensional Poverty Index (MPI), health system coverage, share of home-care services offering PC, opioid consumption per capita, pediatric serious health-related suffering index, Human Development Index (HDI), and number of PC-specific services per population.

### Data sources

Data were obtained from official public sources: the National Administrative Department of Statistics (DANE) [24], the High-Cost Account (CAC) [25], the Ministry of Health open data platform (OTIC) [26], and the Colombian Palliative Care Observatory (OCCP) [27], complemented by peer-reviewed literature and technical reports [2, 12].

Data extraction occurred between1 February and 1 July 2025, using the dataset versions available on those dates. All sources correspond to the most recent 2024 departmental releases. All indicators were extracted from standardized administrative databases, ensuring consistent measurement definitions and full comparability across departments. No measurement procedures differed among territorial units or across data sources.

Detailed definitions, data sources, numerators and denominators, and classification of indicators as reflecting system capacity or contextual need for the eight indicators retained in the final SIPCI are provided in Supplementary Table 1.

### Bias

Multiple strategies were applied to minimize potential biases.

- **Missing data bias:** no missing data were identified. Variable distributions were assessed (most were non-normal), which informed the choice of Min–Max normalization without excluding indicators for this reason.
- **Administrative data bias:** although complete datasets were available, under-registration and measurement error may persist in administrative sources and may vary across departments, potentially leading to underestimation of integration in territories with weaker reporting capacity.
- **Collinearity:** only indicators with Spearman *ρ > 0.82* were removed to reduce redundancy while preserving conceptual coherence; VIF was also examined (e.g., VIF * > 10*).
- **Weighting bias:** robustness analyses used alternative weighting schemes (equal, hierarchical by domain, geometric, and Monte Carlo simulations) to test stability under different assumptions.
- **Geographical coverage bias:** no department was excluded. Indicators available only at national level (no departmental disaggregation) were omitted to avoid structural bias.
- **Equity:** We acknowledge that index construction and weights may influence prioritization of disadvantaged territories; transparency and territorial consistency were explicitly verified.
- **Ecological bias:** given the ecological nature of the study, potential ecological fallacy was acknowledged; no individual-level inference was drawn from aggregated departmental data.

### Ethical considerations

This study complied with the principles of the Declaration of Helsinki. Ethical approval was granted by the Bioethics Committee of the Faculty of Medicine at Antonio Nariño University (Colombia), under Act No 13-2024, dated August 21, 2024. No methodological changes were made after ethics approval.

## Results

### Indicator selection and internal consistency

Descriptive statistics were computed for all 33 territorial units. No missing data were found for any indicator. Fifteen candidate indicators were initially identified through the Delphi process. Eight belonged to the Palliative Care Development Indicators domain (53%), mainly distributed across subdomains such as essential medicines (*n=1*), training and education (*n=4*), and health service delivery/needs estimation (*n=3*). Seven indicators (47%) originated from the PHC Components framework, corresponding to determinants and health burden, service delivery outputs, integrated services (access/equity, effective coverage, and utilization of services), Impact (suffering reduction / avoidable mortality), and multisectoral policy or financial protection. According to Donabedian’s model, these indicators included three structural, two process, and ten outcome measures. Table 1 summarizes the initial classification and those retained for PCA.

**Table 1:** Classification of indicators according to the WHO frameworks for palliative care development and the WHO–UNICEF primary health care monitoring framework, following the Donabedian model

PC Development and PHC framework	Domain and component	Indicators	Donabedian Model	Included in PCA
Palliative Care development indicators	Essential medicines	Availability of 24/7 pharmacies for opioid dispensing	Structure	Yes
	Health service delivery (needs estimation)	Number of specialized palliative care services in the country per population	Structure	Yes
		Proportion of home care services offering palliative care	Process	Yes
	Training and education	Nursing undergraduate programs with specific palliative care training	Structure	Yes
		Index of serious health-related suffering (0–14 years)	Outcome	No
		Medical undergraduate programs with specific palliative care training	Structure	Yes
		Pediatric palliative care training programs for medical professionals	Structure	No
		Pediatric palliative care training programs for nursing professionals	Structure	No
PHC Component	Determinants / Health burden (needs estimation)	Prevalence of chronic diseases	Outcome	Yes
	Service delivery (outputs; utilization of services)	Number of people attended in health services by diagnosis (tumors, circulatory, chronic respiratory diseases, diabetes)	Outcome	Yes
	Integrated services (access / equity)	Access to health services in rural areas	Process	Yes
	Integrated services (effective coverage)	Proportion of the population with access to basic health services	Outcome	Yes
	Impact (suffering reduction / avoidable mortality)	Mortality rate from chronic diseases amenable to palliative care	Outcome	Yes
	Integrated services (appropriate utilization)	Proportion of hospitalizations due to chronic diseases requiring palliative care	Process	Yes
	Multisectoral policy / action (financial protection)	Health insurance coverage rate	Outcome	No

*Legend:* The table presents the conceptual classification of the indicators used in this study according to the domains and components of the WHO frameworks for Palliative Care Development and the WHO–UNICEF Primary Health Care (PHC) Monitoring Framework. Indicators are also categorized according to the Donabedian model (structure, process, outcome). The last column indicates whether each indicator was included in the Principal Component Analysis (PCA)

After internal consistency testing, 11 indicators were retained for PCA. Four indicators were excluded after the analysis due to a combination of sampling adequacy, low individual KMO (* < 0.5*), excessively high Spearman’s correlations (*p = 0.82*), or elevated collinearity (VIF * > 10*). Specifically, the excluded indicators were: (1) Health Insurance Coverage (*p = 0.91*, KMO = 0.41); (2) Pediatric Serious Health-Related Suffering Index, 0–14 years (*p = 0.82*, VIF = 10.76), and (3–4) Training Programs in Pediatric Palliative Care for Nursing and Medical Professionals (both *p = 1*, VIF = *∞*).

After the exclusion of four indicators, overall sampling adequacy improved from KMO = 0.699 (15 indicators, “regular”) to KMO = 0.742 (11 indicators, “adequate”), after removing these four, increasing representing an increase of approximately 6%. Among retained indicators, no pairwise correlation exceeded *ρ = 0.82*; individual KMO values averaged 0.647, and most VIF values were below 6, with only one reaching 10.7. These results indicate acceptable internal consistency and manageable multicollinearity within the final set of indicators, confirming their suitability for PCA (Table 2).

**Table 2:** Assessment of internal consistency, collinearity, and exclusion criteria for indicator selection

Indicator	KMO	Correlation	VIF	Exclusion decision
Access to health services in rural areas	0.48	0.91	4.87	No
Health insurance coverage	0.41	0.91	6.24	Yes
Availability of 24/7 pharmacies for opioid dispensing	0.68	—	16.05	No
Hospitalizations due to chronic diseases)	0.53	—	1.42	No
Index of serious health-related suffering (age 0–14)	0.63	0.82	10.76	Yes
Mortality from chronic diseases amenable to palliative care	0.82	—	4.33	No
Number of people attended in health services by diagnosis	0.71	0.82	32.86	No
Number of specialized palliative care services per population	0.22	—	2.42	No
Prevalence of chronic diseases	0.58	—	1.94	No
Nursing programs with specific palliative care training (undergraduate)	0.78	1.00	*∞*	No
Pediatric palliative care training for nurses	0.78	1.00	*∞*	Yes
Pediatric palliative care training for physicians	0.72	1.00	51.33	Yes
Medical programs with specific palliative care training (undergraduate)	0.74	1.00	82.08	No
Proportion of population with access to health services	0.60	—	2.38	No
Proportion of home care services offering palliative care	0.91	—	5.89	No

*Legend:* This table shows the statistical evaluation of each indicator using the Kaiser–Meyer–Olkin (KMO) test for internal consistency, the correlation coefficient with other indicators, and the Variance Inflation Factor (VIF) for collinearity. The final column reports whether the indicator was excluded from the PCA based on internal consistency, collinearity statistics, and conceptual considerations

### Principal component analysis (PCA)

**Fig. 2:**
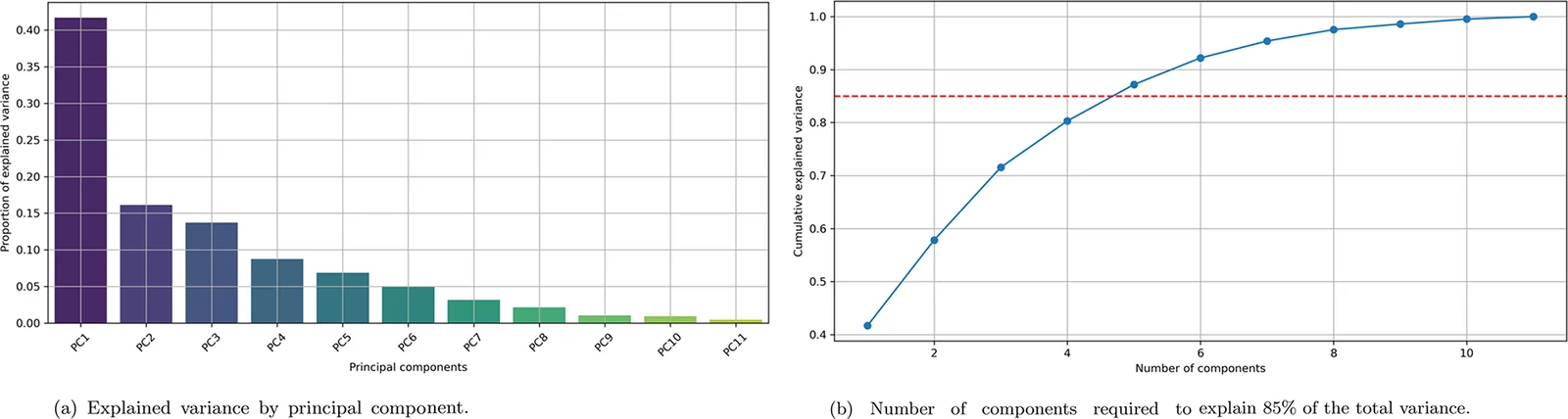
Principal component analysis (PCA) of the selected indicators. *Legend:* The scree plot (**a**) and cumulative variance plot (**b**) show that five principal components explain 85% of the total variance, meeting the predefined threshold for dimensionality reduction

Figure 2 shows the PCA results. The first component (PC1) explained slightly over 40% of total variance, followed by PC2 (15%). Five components were required to reach the cumulative 85% variance threshold. The mean normalized mean squared error (NMSE) across indicators was 0.13, indicating that approximately 87% of the original information was preserved in the reduced-dimensional space. Supplementary Table 2 presents the normalized indicator matrix and the principal-component scores for all departments and the capital district.

### Varimax rotation and indicator representativeness

Varimax rotation identified representative indicators for each component using the 85th percentile of the absolute rotated loadings ($|\lambda| \geq 0.426$) as the selection threshold. Only one indicator, Proportion of Population with Access to Health Services, exceeded this threshold in two components (PC2 = 0.588, PC4 = 0.485); following OECD guidelines, it was assigned to the component with the higher loading to avoid double-weighting.

The highest loading was for Access to Health Services in Rural Areas (PC3 = 0.932), followed by Hospitalizations for Chronic Conditions (PC5 = 0.904). Three indicators loaded on PC1, two on PC2, and one each on PC3, PC4 and PC5. Supplementary Table 3 presents the rotated loading matrix for all indicators across the five retained components. Supplementary Table 4 summarizes the representative indicators selected per component according to the 85th percentile threshold

A reduced PCA (eight indicators) was performed to confirm internal validity. The NMSE decreased from 0.13 to 0.10 (10%), confirming lower information loss and high fidelity. This indicates that 90% of the information was preserved after reduction. Communality values ($h_i^2$) for all retained indicators exceeded 0.7, confirming that each contributed substantially to the underlying construct of PC integration into PHC. The indicator Access to Health Services in Rural Areas had the highest communality (0.99), followed by Undergraduate Medical Training in PC (0.91), while Availability of 24/7 pharmacies for opioid dispensing had the lowest (0.80). A reconstruction test comparing the PCA models (11 vs. 8 indicators) yielded an NMSE of 0.12, corresponding to 88% information retention. Together, these results confirmed that dimensionality reduction produced a stable, low-loss representation of the system. All NMSE values remained below 0.2, which is considered low and acceptable for composite indicator modeling. These diagnostics indicate that even the least well-represented indicator retains a high proportion of shared variance, supporting confidence in all retained indicators and justifying the stability of the latent structure underlying the eight-indicator SIPCI. Supplementary Figure 1 displays the individual communality and normalized mean squared error values for each indicator, confirming strong representation and minimal information loss after dimensionality reduction.

### Construction of the synthetic index of palliative care integration into primary health care (SIPCI)

**Fig. 3:**
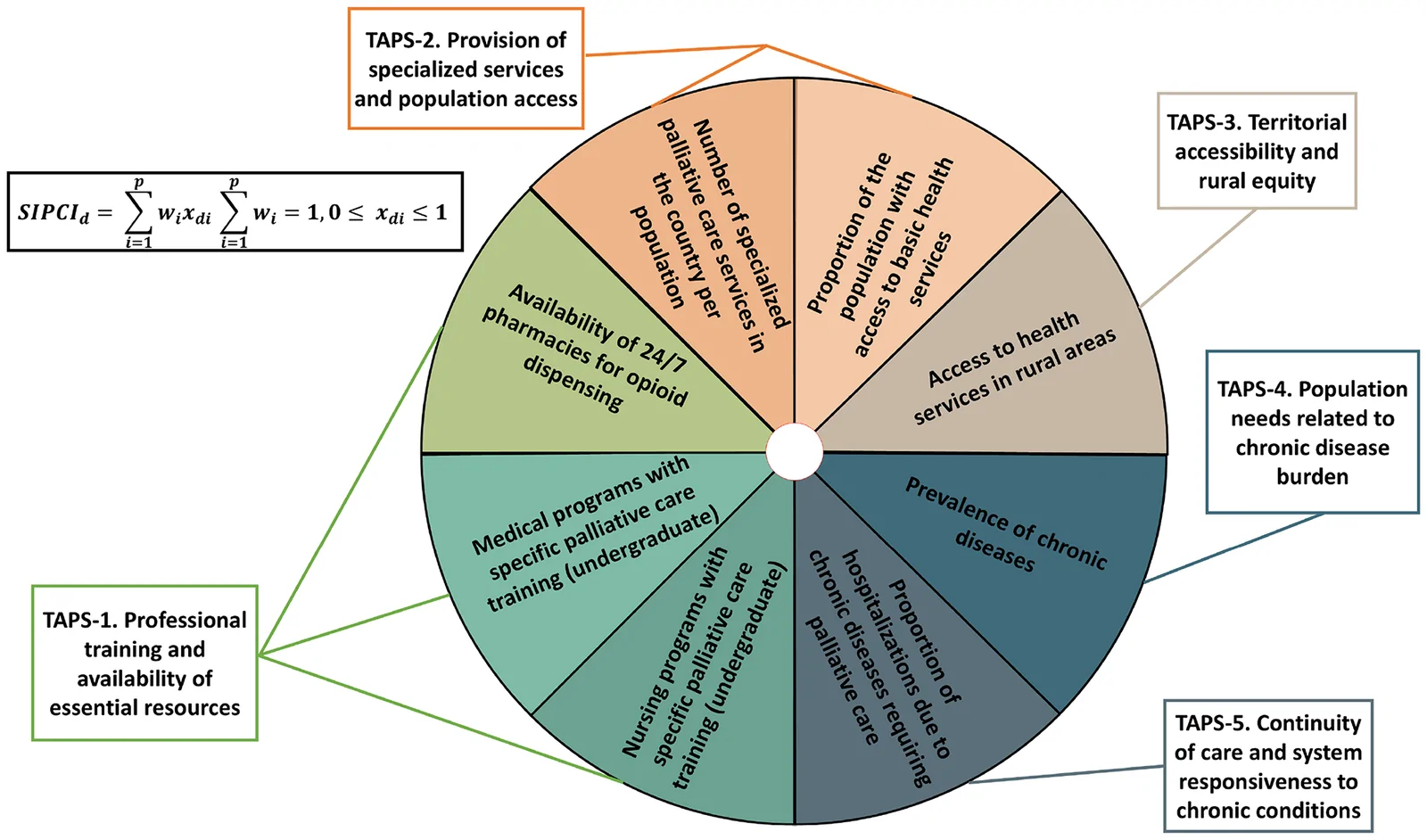
Structure and conceptual components of the SIPCI index. *Legend:* The eight indicators included in the SIPCI are grouped into five principal components (TAPS). The mathematical expression for weighted aggregation appears in the upper left. Colors represent conceptual domains derived from PHC principles

Indicators were grouped and named according to their thematic alignment with Primary Health Care, referred to as TAPS (Terms Aligned with PHC), as shown in Fig. 3. Each TAPS corresponds to one rotated component identified in the PCA.

**TAPS-1 (Training and essential resources): Professional training and essential resources.** Reflects the availability of human resources trained in palliative care and access to essential medicines. **TAPS-2 (Specialized service provision and population access): Provision of specialized services and population access.** Captures the supply of PC specific services and overall access to health care. **TAPS-3 (Rural access and territorial equity): Rural accessibility and territorial equity.** Represents geographical disparities in access to health services. **TAPS-4 (Chronic disease burden and population need)**: Population needs related to chronic disease burden. Reflects the magnitude of chronic conditions requiring palliative approaches. **TAPS-5 (Continuity of care and system responsiveness): Continuity of care and system responsiveness.** Indicates the system’s capacity to respond to chronic disease burden, even through hospital-based care.

Table 3 presents the fixed weights derived from each component’s explained variance and squared loadings. The indicator with the largest contribution to SIPCI was Access to Health Services in Rural Areas (TAPS-3; 17%), followed by Undergraduate Nursing Training in PC (TAPS-1; 16%) and Number of PC-Specific Services per Population (TAPS-2; 13%). The lowest contributions were Proportion of hospitalizations due to chronic diseases requiring palliative care (TAPS-5; 9%) and Proportion of Population with Access to Health Services (TAPS-2; 8%).

**Table 3:** Final weights of indicators for the Synthetic index of palliative care integration (SIPCI) by TAPS domain

TAPS	Indicator	Loading	s_ik_	W_dom_	W_final_	W_final%_
TAPS-1. Professional training and availability of essential resources	Nursing programs with specific palliative care training (undergraduate)	0.492	0.395	0.407	0.161	16.063
	Medical programs with specific palliative care training (undergraduate)	0.436	0.309	0.407	0.126	12.576
	Availability of 24/7 pharmacies for opioid dispensing	0.427	0.296	0.407	0.121	12.067
TAPS-2. Provision of specialized services and population access	Number of specialized palliative care services in the country per population	0.786	0.641	0.208	0.133	13.328
	Proportion of the population with access to basic health services	0.588	0.359	0.208	0.075	7.464
TAPS-3. Territorial accessibility and rural equity	Access to health services in rural areas	0.932	1.000	0.172	0.172	17.199
TAPS-4. Population needs related to chronic disease burden	Prevalence of chronic diseases	0.809	1.000	0.122	0.122	12.177
TAPS-5. Continuity of care and system responsiveness to chronic conditions	Proportion of hospitalizations due to chronic diseases requiring palliative care	0.904	1.000	0.091	0.091	9.127

*Legend:* $\mathbf{s_{ik}}$ = relative contribution of indicator i within domain k; $\mathbf{W_{dom}}$ = weight assigned to the domain; $\mathbf{w_{final}}$ = final weight of the indicator after applying the corresponding domain weight

The weight distribution was balanced across domains, supporting the multidimensional nature of the index. Although Access to Health Services in Rural Areas showed the largest individual contribution, SIPCI should not be interpreted as a measure of rural accessibility alone. Rather, rural accessibility represents one of several complementary dimensions of palliative care integration within primary health care. The final index combines indicators reflecting workforce preparedness, service availability, territorial accessibility, population needs, and system responsiveness, Consequently, SIPCI is intended to reflect the overall degree of palliative care integration within PHC systems rather than the performance of any single domain.

Domain weights reflect the relative contribution of each latent dimension to the overall SIPCI, based on the proportion of variance explained by the corresponding principal component. Within each domain, indicator weights represent the relative influence of each indicator within that dimension, derived from the squared rotated loadings and normalized so that weights sum to one. This hierarchical weighting approach preserves the multidimensional structure of palliative care integration while improving transparency and interpretability for health system users.

#### How to interpret SIPCI scores

SIPCI values range from 0 to 1 and reflect relative levels of palliative care integration into primary health care at the departmental (system) level, based on the eight indicators and their fixed weights. Values closer to 1 indicate higher observed integration relative to other departments, whereas lower values indicate a position at the lower end of the national distribution; importantly, they do not imply the absence of palliative care services. No department reached the theoretical maximum value of 1, indicating that none simultaneously achieved the highest observed values across all indicators. For descriptive purposes, departments were grouped into quartiles (Q1–Q4), which represent relative positions within the national distribution rather than absolute performance thresholds. As a reference, a difference of 0.10 in SIPCI corresponds to a 10-percentage-point change on the index scale and should be interpreted as a meaningful shift in the combined weighted indicator profile, particularly when changes occur in higher-weight dimensions.

### Departmental results of the SIPCI index

**Fig. 4:**
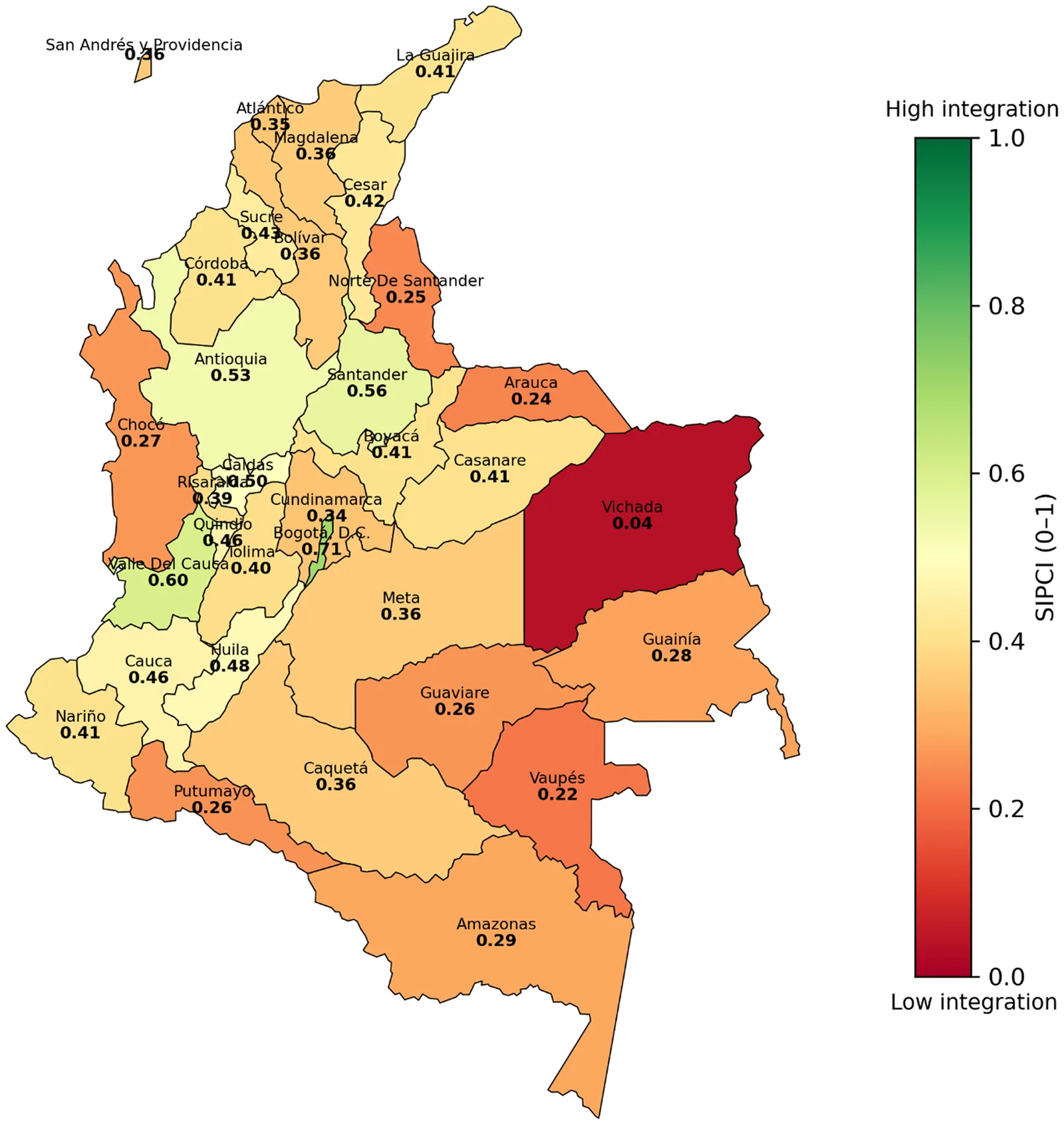
Synthetic index of palliative care integration into primary health care (SIPCI) by department, Colombia 2024. *Legend:* Departmental SIPCI scores classified by quartiles. Warm colors (red–orange) indicate lower integration; cooler tones (light yellow to green) represent higher integration

After weighted linear aggregation, SIPCI values ranged from 0.04 to 0.71 (median = 0.39, mean = 0.38, SD = 0.13). Figure 4 presents the spatial distribution of SIPCI across Colombia. To facilitate interpretation, departments were classified into four integration levels based on quartile thresholds: low integration (*≤ 0.291*), medium-low integration (0.291–0.395), medium-high integration (0.395–0.434), and high integration (* > 0.434*). No department reached the maximum value (1.0). Approximately 48% of departments were classified as “high” or “medium-high” integration, while 52% were “low” or “medium-low”. Lower integration was observed toward the geographic periphery of the country. Exact SIPCI values and standardized indicator scores for all departments are provided in Supplementary Table 5.

**Fig. 5:**
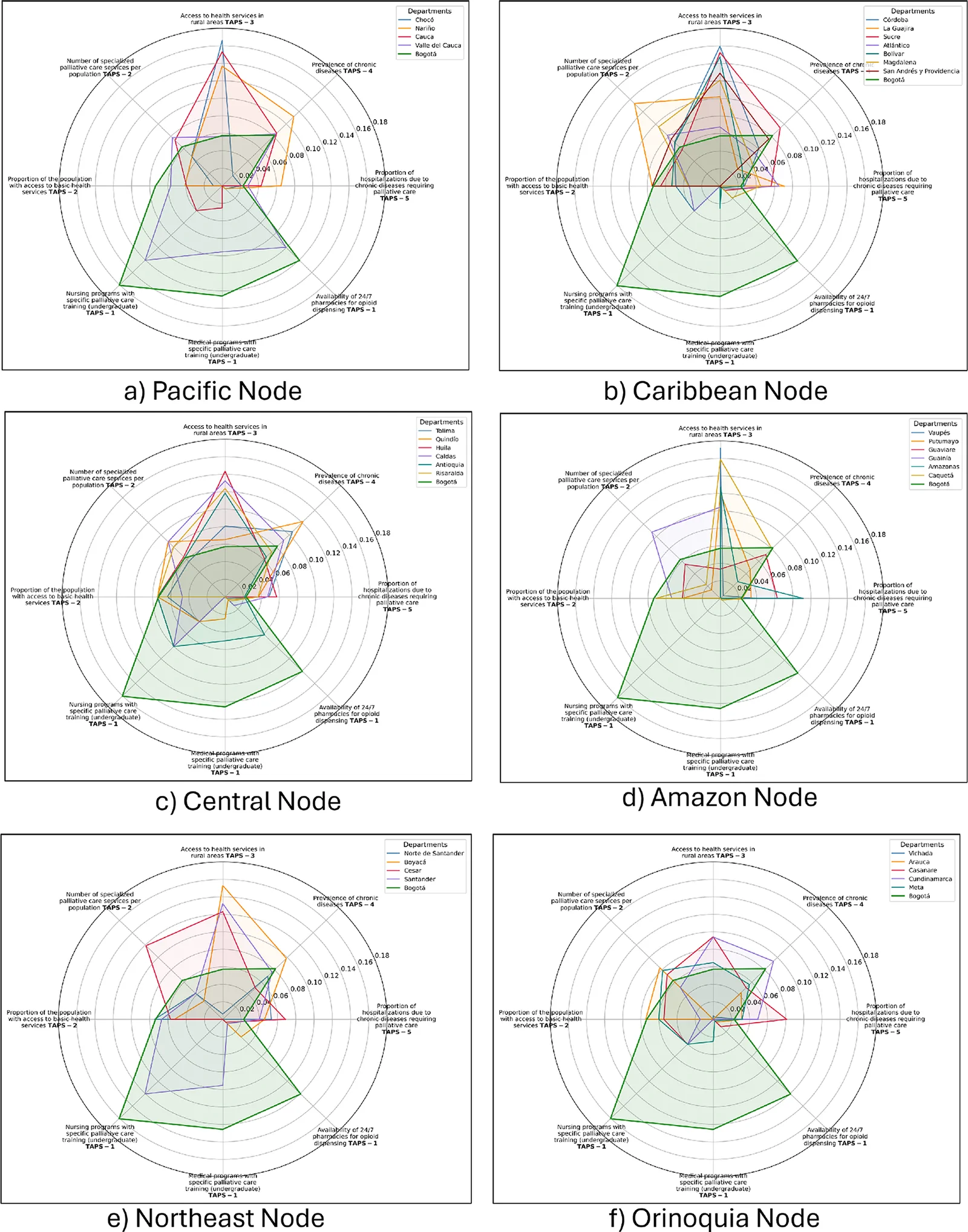
Radar profiles of SIPCI indicators by regional node. *Legend:* Radar charts showing SIPCI index profiles by regional node. Each panel represents one of the seven geographic and functional nodes., comparing departments within each node. The eight axes correspond to the SIPCI index, grouped by TAPS dimensions (TAPS-1 to TAPS-5). Bogotá (green) is shown as a reference in all panels

To further explore regional patterns, departments were grouped into the seven geographic and functional nodes (regional clusters) proposed by León et al. [23]. (Fig. 5). The 32 departments and one special administrative unit were distributed across these nodes.

Lower integration levels were concentrated in the peripheral parts of the country, particularly in the Amazon node (e.g., Vaupés 0.22, Amazonas 0.29, Guainía 0.28, Guaviare 0.26), followed by the Orinoquia node (e.g., Vichada 0.04, Arauca 0.24). Higher levels of integration were observed in the Central and Northeast nodes, as well as in the Bogotá node (e.g., Bogotá 0.71, Antioquia 0.53, Huila 0.48, Santander 0.56).

In the Caribbean and Pacific nodes, departments were distributed across all integration categories: high (Valle del Cauca 0.60), medium-high (La Guajira 0.41), medium-low (San Andrés and Providencia 0.36), and low (Chocó 0.27). Overall, the results reveal a clear spatial gradient in SIPCI values, with higher integration concentrated in central regions and lower integration toward the geographic periphery of the country.

### Radar profiles by integration quartiles

**Fig. 6:**
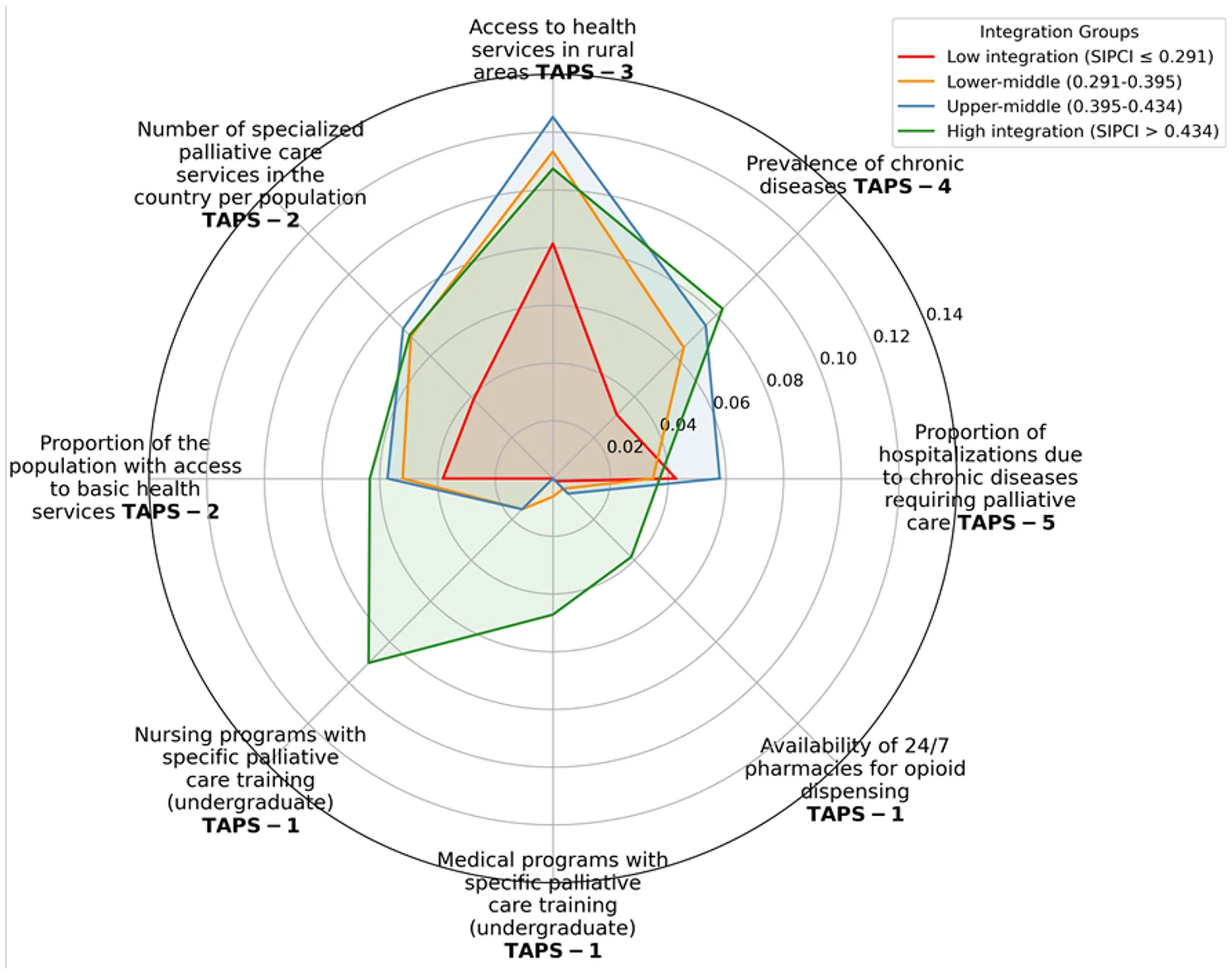
Radar profile of SIPCI index by integration quartile. *Legend:* Average standardized indicator values for departments within each integration quartile. Lines represent quartile groups (Q1–Q4); each axis corresponds to one of the eight SIPCI indicators

The radar plot (Fig. 6) shows the average profile of the four SIPCI integration levels across the eight indicators. Non-monotonic patterns across quartiles are expected in multidimensional integration measures. The five TAPS dimensions are not hierarchical and may evolve asynchronously: a department may score relatively high in specialized service provision or hospital-based responsiveness while remaining constrained in workforce training, essential resources, or rural accessibility. In addition, because SIPCI includes both capacity-related and need-related indicators, higher need can coexist with higher observed integration signals without implying better outcomes. Therefore, radar profiles should be interpreted as domain-specific configurations that highlight strengths and gaps, rather than as a uniform stepwise progression across all dimensions. Although patterns were not strictly monotonic, consistent gradients emerged across domains. Departments in the “low integration” group scored near zero for TAPS-1. Professional training and essential resources, while those in the “high integration” group achieved notably higher values. Conversely, the “medium-high” group outperformed even the “high” group in TAPS-2. Provision of specialized services and population access, and TAPS-3. Rural accessibility and equity, indicating regional variability in service distribution. TAPS-4. Population needs related to chronic disease burden followed a consistent gradient, with higher values for higher integration groups. TAPS-5. Continuity of care and system responsiveness showed its highest average in the “medium-high” group, suggesting that hospital-based responsiveness may not linearly align with overall integration level Supplementary Table 5.

### Sensitivity, robustness, and uncertainty analyses

The leave-one-out (Jackknife) sensitivity analysis indicated strong stability of SIPCI. When each indicator was removed in turn and weights were re-normalized, correlations with the base index remained very high (Spearman = 0.96; Kendall’s $\tau_b = 0.86$; mean absolute rank error [MAE] low (1.81)). The least influential indicators were 24/7 Pharmacies Dispensing Opioids (Spearman = 0.99; Kendall = 0.94; MAE = 0.91) and Proportion of Population with Access to Health Services (Spearman = 0.98; Kendall = 0.89; MAE = 1.33). The most influential was Access to Health Services in Rural Areas (Spearman = 0.89; Kendall = 0.72; MAE = 3.51). Overall, the findings confirmed that no single indicator dominated the structure of SIPCI and that the index remained stable to individual exclusions Supplementary Table 6.

Alternative normalization methods (z-score, winsorized Min–Max, robust Min–Max, median/IQR, and rank scaling) showed high consistency with the baseline SIPCI (Spearman * > 0.94*). The IQR, and rank-based variants presented slightly higher ranking errors (MAE = *2.36* and *2.67*, respectively), while z-score, winsorized, and robust Min–Max maintained excellent stability (MAE * < 1.1*) Supplementary Table 7.

Four alternative weighting scenarios further confirmed structural robustness: R1: Equal weights, high stability (*ρ = 0.98*, MAE = 1.58); R2: Equal weights by domain with intra-domain allocation by squared loadings, moderate stability (*ρ = 0.92*, MAE = 2.73); R3: Geometric aggregation weighted by explained variance, lower stability (*ρ = 0.83*, MAE = 3.75); R4: Monte Carlo simulation ($\pm 20\%$ perturbation, *n = 1000*), median *ρ = 0.99* (p5–p95 = [0.98–1.00]) Supplementary Table 8. These results confirmed that SIPCI was robust to alternative assumptions regarding weighting and normalization.

Across plausible methodological choices, including indicator exclusion, alternative normalization, weighting schemes, and Monte Carlo perturbations, departmental rankings changed minimally, indicating that SIPCI is unlikely to be an artifact of any single analytic decision.

### External validity

To guide interpretation of the external validation analyses, external variables were conceptually grouped into two categories. Capacity-related validators (e.g., opioid availability and share of home-care services offering palliative care) were interpreted as proxies of service availability and health system capacity for delivering palliative care. In contrast, context- or need-related validators (e.g., poverty indices, suffering indicators, and number of patients with palliative-relevant diagnoses) were interpreted as reflecting underlying population needs and disease burden rather than higher-quality or better-performing integration. General socioeconomic indicators (GDP per capita, Human Development Index, and overall health system coverage) were treated as contextual benchmarks that characterize the broader development environment in which palliative care integration occurs, rather than as direct measures of palliative care capacity or need.

All associations were estimated with Pearson’s correlation coefficient *r*. SIPCI showed a strong positive association with GDP per capita (*r = 0.62*, *p < 0.000*) and strong negative association with the MPI (*r = -0.64*, *p < 0.000*), indicating that higher levels of palliative care integration are more frequently observed in departments with greater economic development and lower poverty. In contrast, the association with overall health system coverage was weak and non-significant (*r = 0.25*, *p = 0.155*). The correlation with the HDI was also weak and non-significant (*r = 0.19*, *p = 0.296*), suggesting that general development indicators alone do not capture specific service capacities required for palliative care integration (Supplementary Table 9).

Regarding capacity-related service indicators, SIPCI showed a strong positive association with opioid availability (*r = 0.70*, *p < 0.000*) and with the proportion of home-care services offering palliative care (*r = 0.68*, *p < 0.000*), supporting the interpretation that higher SIPCI values reflect better access to essential palliative care services.In contrast, the association with the number of specialized palliative care services per population was weaker (*r = 0.39*, *p < 0,026*), suggesting that numerical service availability alone does not fully capture territorial or functional integration.

For context- and need-related validators, SIPCI showed a strong positive association with the number of patients attended for palliative-relevant diagnoses (*r = 0.71*, *p < 0.000*) and a moderate positive association with the pediatric serious health-related suffering index (*r = 0.58*, *p < 0.000*). Given that higher values of these indicators represent greater burden or suffering, these associations were interpreted as reflecting alignment between higher levels of integration and greater underlying need, rather than improved outcomes. All estimates were unadjusted correlations, as no individual-level confounding factors were applicable in this ecological design.

## Discussion

This study aimed to design and validate a new composite indicator, the SIPCI Index of Palliative Care Integration into Primary Health Care, to assess the degree of integration of PC within the PHC system in the departments of Colombia in 2024. The methodological process followed international guidelines for developing composite indicators [14, 28–30], including internal consistency tests, PCA with varimax rotation, and extensive sensitivity and robustness analyses. The final model showed good adequacy (KMO = 0.742) and preserved nearly 90% of the original information (NMSE *≈ 0.10*), confirming internal coherence and parsimony. High Spearman (* > 0.94*) and Kendall’s (* > 0.86*) coefficients confirmed the structural stability of the index [31, 32].

About 52% (17 of 33) of departments were classified as having low or medium-low integration, and 48% (16 of 33) as medium-high or high. Integration showed a marked spatial gradient: central regions and Bogotá scored highest, while Amazon and Orinoquia departments remained lowest. These disparities mirror the territorial inequities already reported in Colombia’s health-service distribution [23, 33].

The positive but moderate correlation between SIPCI and departmental GDP per capita (*r* = 0.62) should be interpreted cautiously. GDP reflects productive capacity, not the allocation or efficiency of health expenditure. Economic development may facilitate integration but does not ensure it. These findings suggest that the translation of financial resources into effective service integration depends on governance, workforce distribution, and intersectoral coordination. Several departments with intermediate GDP; such as Cundinamarca, Atlántico, and Arauca, had limited integration, suggesting that governance, workforce distribution, and service coordination matter more than macroeconomic wealth alone. Conversely, departments with both low GDP and low SIPCI, including Vichada and Guainía, combine economic and institutional barriers [24, 34, 35].

These findings are consistent with the interpretation that economic capacity may facilitate palliative care integration, but it does not reliably predict it. Differences observed across departments may reflect variation in governance, workforce distribution, and service organization, which SIPCI captures descriptively at the system level. In this context, existing national legislation and regulatory frameworks provide a reference, but the degree of integration observed across territories remains heterogeneous [27, 36, 37]. At the policy level, SIPCI can help identify domains where implementation appears weaker, such as workforce training or rural accessibility, and can inform prioritization and follow-up within existing regulatory frameworks.

From a public-health perspective, these results align with the WHO position that the integration of PC into PHC is essential to achieve UHC and continuity of care [2]. The SIPCI captures this multidimensional nature through its five thematic axes (TAPS): training and resources (TAPS-1), service availability and access (TAPS-2), rural accessibility (TAPS-3), chronic-disease needs (TAPS-4), and continuity and responsiveness (TAPS-5). The indicator “Access to health services in rural areas,” which showed the highest communality (0.99), emphasizes the importance of territorial accessibility as a structural condition for integration rather than a direct measure of equity. Together, these dimensions portray integration as a systemic rather than a purely clinical phenomenon [2, 12, 38].

At the same time, it is important to distinguish between statistical validity and construct validity. The analyses conducted in this study provide evidence of internal coherence, dimensional structure, and robustness under alternative weighting and normalization scenarios. However, these findings alone do not fully establish construct validity. As highlighted in the OECD Handbook on Constructing Composite Indicators, statistical techniques can support indicator selection, weighting, and dimensionality reduction, but the conceptual validity of a composite index also depends on the theoretical framework underlying its construction [14]. In the present study, construct validity was supported through the use of internationally recognized frameworks for palliative care and primary health care, a structured Delphi process, self-organizing maps, and complementary qualitative analysis of expert perspectives [15, 16]. Taken together, these conceptual and empirical procedures support the interpretation of SIPCI as a measure of palliative care integration within primary health care. Nevertheless, integration remains a multidimensional and context-dependent concept, and future studies should continue examining the performance of SIPCI in other settings and against additional external criteria.

Education and workforce capacity emerged as critical drivers. Undergraduate training in PC for nursing and medical students contributed substantially to SIPCI performance, confirming evidence that early inclusion of PC in general curricula strengthens PHC responsiveness and community care [39–42]. In Latin America, integration efforts remain fragmented; PC services often coexist with PHC programmes but are rarely functionally aligned [8, 43]. Strengthening professional competencies at the primary-care level is therefore a realistic entry point for advancing integration across the region [44, 45].

Service availability also influences integration. The inclusion of “home-care services offering palliative care” shows that first-level service capacity contributes to functional integration. However, the indicator “hospitalizations for chronic conditions” aggregates all levels of care, which limits its interpretability. For this initial analysis, it is considered a proxy indicator suggesting that, at a minimum, people with chronic conditions who require health care are in contact with the health system, rather than a direct measure of integrated or high-quality care [46–48]. Future analyses should examine patient-level trajectories to assess whether higher integration correlates with fewer preventable hospital admissions. This aligns with the notion that integrated networks, rather than isolated services, drive continuity across levels of care, as also emphasized in the PAHO Strategic Plan on resilient health systems based on primary health care [48, 49]. When patients can move seamlessly among home-based, ambulatory, and hospital care, the system demonstrates the degree of integration that palliative care requires to be both timely and equitable. The SIPCI thus offers an initial framework to identify territorial differences in service organization and to guide future monitoring of integration across levels of care.

Beyond service organization, system financing and financial protection also shape integration. According to the World Bank, out-of-pocket health expenditure in Colombia represented 15% of current health expenditure in 2023 [50]. Values approaching the 20% threshold identified by WHO as high financial risk level [51] suggest growing pressure on households and potential barriers to effective access. When families must assume increasing direct costs, the capacity of PHC services to deliver integrated palliative approaches is undermined. These financial and organizational barriers converge to explain why economic capacity alone does not translate into integration. This evidence situates the SIPCI within a broader context where financial protection is declining despite nearly universal insurance coverage.

Departments with relatively high GDP but modest SIPCI scores, such as Atlántico or Cundinamarca, likely reflect persistent fragmentation: hospital-centred models, weak coordination between service levels, and limited implementation of national PC policies. These challenges are consistent with previous analyses showing that palliative care in Colombia and Latin America remains concentrated in urban tertiary settings rather than embedded within PHC [5]. The SIPCI captures how such structural gaps translate into measurable disparities.

From a policy perspective, findings suggest that improving integration is likely to depend on the operationalization of existing national PC legislation through effective implementation within PHC programmes, particularly in rural areas. Priorities include workforce development, equitable distribution of essential resources, and monitoring of integration progress through composite metrics such as the SIPCI. Because national policies already define regulatory frameworks, departmental actions should focus on execution and coordination rather than new legislation [47].

The SIPCI is intended as a system-level monitoring tool. It can be used to support routine follow-up of palliative care integration within primary health care, to identify territorial gaps in domains such as workforce training, rural accessibility, and service organization, and to inform the implementation of national palliative care policies at the departmental level. SIPCI is not a patient outcome index, not a hospital performance score, and it is not intended for causal inference. It provides a descriptive view of integration and system responsiveness at the health-system level, rather than a measure of individual-level quality of care.

From an implementation perspective, SIPCI can be used by departments to identify which system domains are most strongly associated with lower levels of palliative care integration. By examining domain-specific contributions, decision-makers can distinguish whether gaps are primarily driven by workforce training, rural accessibility, service availability, or system responsiveness, and can prioritize context-appropriate actions within existing policy frameworks. For example, low scores in training-related domains may support expanding undergraduate palliative care curricula, while deficits in rural accessibility may highlight the need to strengthen outreach pathways or service coverage outside urban centers.

The practical applicability of SIPCI is supported by several facilitators, including the use of existing administrative data sources, open access to data and code, and stable weighting derived from internal structure rather than normative assumptions. At the same time, potential barriers should be acknowledged, such as uneven data quality or under-registration across territories, limited subnational granularity for some indicators, and the risk of misinterpretation if the index is used as a punitive ranking rather than as a descriptive monitoring tool.

### Limitations

This study has limitations. Being ecological and cross-sectional, causal inferences cannot be made. No missing data were detected, although under-registration of some indicators may persist.

Nevertheless, sensitivity and robustness analyses confirmed both internal and external consistency. Some indicators, such as hospitalizations for chronic diseases and the number of patients attended, should be interpreted with caution. Their positive correlation with SIPCI likely reflects greater demand and need for care rather than higher integration or performance. In this first measurement of palliative care integration, hospitalizations were used as a proxy for system contact and service demand; however, future studies should incorporate disaggregated data to differentiate home-based, ambulatory, and inpatient care. Tracking SIPCI scores over time and linking them to patient-level outcomes, such as hospital readmissions, symptom control, or place of death, could further strengthen its interpretive validity.

Correlations used for external validation were exploratory and were interpreted primarily in terms of magnitude and theoretical coherence, rather than formal hypothesis testing.

Although multiple procedures were used to support the conceptual basis of SIPCI, including international frameworks, expert consensus, self-organizing maps, and qualitative analysis, the extensive statistical validation performed in this study does not by itself guarantee that the index fully captures all dimensions of palliative care integration. As with other composite indicators representing complex health-system constructs, SIPCI should be interpreted as an evidence-informed operationalization of integration rather than as an exhaustive representation of the concept. Further studies in different contexts and using additional external criteria may help strengthen its construct validity.

The transferability of the SIPCI to other countries depends on the availability of comparable subnational indicators and on contextual adaptation of domains and weights. Differences in health system organization, data infrastructure, and palliative care policy frameworks may limit the direct application of the index without contextual calibration.

In addition to these data-driven extensions, future research may complement this system-level approach by examining contextual and operational factors that affect the implementation of palliative care, such as facility-level constraints, management of controlled medicines, and ethical considerations related to resource allocation, using qualitative or policy-oriented study designs.

## Conclusions

The SIPCI provides an operational and reproducible monitoring and benchmarking instrument, offering moderate-strength evidence for system-level assessment of palliative care integration into primary health care at the departmental level in Colombia. The results describe marked territorial heterogeneity, indicating that higher levels of economic development are not consistently associated with higher levels of measured integration.

SIPCI should not be interpreted as a measure of clinical quality, patient outcomes, or causal impact. Its primary value lies in supporting routine monitoring, identifying territorial gaps, and informing planning and follow-up across specific system domains, such as workforce training, access to essential medicines, service availability, and rural accessibility.

By making multidimensional patterns of integration visible at the system level, SIPCI offers a transparent tool to support health system analysis and policy discussion within existing regulatory frameworks. Future research may complement this approach by examining patient-level trajectories and outcomes using designs suited for causal inference.

Although the results support the internal coherence, robustness, and practical applicability of SIPCI, further research is needed to continue examining its construct validity and performance in other settings. As with other composite indicators representing complex health-system constructs, SIPCI should be interpreted as an evidence-informed operationalization of palliative care integration rather than as its only possible representation. Future applications in different contexts may help refine and strengthen its interpretive validity.

## Electronic supplementary material

Below is the link to the electronic supplementary material.

Supplementary Material 1
**Supplementary Table 1**. Indicator dictionary for the eight indicators included in the SIPCI. The table reports indicator definitions, data sources, numerators and denominators, release year, and classification as reflecting system capacity or contextual need.

Supplementary Material 2
**Supplementary Table 2**. Normalized indicator matrix and principal-component scores for the 33 territorial units of Colombia (2024). Each row represents one department or the capital district. Columns correspond to the eleven normalized indicators (values scaled to *[0,1]*, polarity aligned as “higher = better”). The last eleven columns (PC1–PC11) show the standardized scores from the unrotated principal component analysis. No missing data were identified.

Supplementary Material 3
**Supplementary Table 3**. Rotated loading matrix of the eleven indicators across the five retained principal components. The table displays Varimax-rotated loadings (*λ*) for each indicator. Values in bold indicate the component where each indicator has its highest loading. The 85th percentile threshold ($|\lambda| \geq 0.426$) was used to identify representative indicators.

Supplementary Material 4
**Supplementary Table 4**. Representative indicators per principal component according to the 85th percentile threshold. This table lists the eight indicators with loadings equal to or above *|λ| = 0.426*, selected as representative of each component after Varimax rotation. Each indicator is associated with its corresponding principal component and loading value.

Supplementary Material 5
**Supplementary Table 5**. Departmental SIPCI scores and normalized indicator values (2024). The table reports the normalized values (*[0,1]*) of the eight indicators included in the Synthetic Index of Palliative Care Integration (SIPCI) and the final SIPCI score for each of Colombia’s 32 departments and the capital district.

Supplementary Material 6
**Supplementary Table 6**. Leave-one-out (Jackknife) sensitivity analysis for the SIPCI index. Each row shows the results obtained after removing one indicator at a time and re-normalizing the weights. The table reports Spearman’s and Kendall’s correlations with the baseline SIPCI and the mean absolute ranking error (MAE).

Supplementary Material 7
**Supplementary Table 7**. Comparison of alternative normalization methods for the SIPCI index. The table reports Spearman’s correlations and mean absolute ranking errors (MAE) between the baseline SIPCI and versions computed using different normalization techniques.

Supplementary Material 8
**Supplementary Table 8**. Results of structural robustness scenarios for the SIPCI index. The table presents Spearman’s correlations, percentage of quartile changes, and mean absolute ranking errors (MAE) between the baseline SIPCI and four alternative weighting or aggregation scenarios.

Supplementary Material 9
**Supplementary Table 9**. Pearson correlations between the SIPCI index and external validation variables. The table reports the number of departments (N), Pearson’s correlation coefficients (*r*), and corresponding *p*-values for each external variable tested against the Synthetic Index of Palliative Care Integration (SIPCI). All estimates were unadjusted, given the ecological design of the study.

Supplementary Material 10
**Supplementary Figure 1**. Communalities and normalized reconstruction errors by indicator after Varimax rotation. The figure displays the communality (upper chart) and the normalized mean squared error (lower chart) for each indicator included in the Synthetic Index of Palliative Care Integration (SIPCI). Dashed red lines represent the operational thresholds of 0.7 for high shared variance and 0.2 for acceptable reconstruction error. All indicators exceeded the communality threshold and remained below the error threshold, confirming strong representation and minimal information loss after dimensionality reduction.

## Data Availability

All data and study materials are available in the Supplementary Information files and in the Mendeley Data repository (DOI: https://doi.org/10.17632/dg8btkknvj.1.

## Materials availability

All supplementary materials described in this article are also available in the Mendeley Data repository (DOI: https://doi.org/10.17632/dg8btkknvj.1.

## Code availability

All analysis code used in this study is openly available in the Mendeley Data repository under the same (DOI: https://doi.org/10.17632/dg8btkknvj.1.
